# Common mental disorders in TB/HIV co-infected patients in Ethiopia

**DOI:** 10.1186/1471-2334-10-201

**Published:** 2010-07-09

**Authors:** Amare Deribew, Markos Tesfaye, Yohannes Hailmichael, Ludwig Apers, Gemeda Abebe, Luc Duchateau, Robert Colebunders

**Affiliations:** 1 Department of Epidemiology, Jimma University, Jimma, Ethiopia; 2 Department of Epidemiology and Social Sciences, Antwerp University, Antwerp, Belgium; 3 Department of psychiatry, Jimma University, Jimma, Ethiopia; 4 Department of Health Service management, Jimma University, Jimma, Ethiopia; 5 Institute of Tropical Medicine, Antwerp, Belgium; 6 Department of Medical Laboratory Sciences and Pathology, Jimma University, Jimma, Ethiopia; 7 Department of Physiology and Biometrics, Ghent University, Ghent, Belgium

## Abstract

**Background-:**

The relationship between TB/HIV co-infection and common mental disorders (CMD) has been scarcely investigated. In this study, we compared the occurrence of CMD in TB/HIV co-infected and non-co-infected HIV patients in Ethiopia.

**Methods-:**

We conducted a cross sectional study in three hospitals in Ethiopia from February to April, 2009. The study population consisted of 155 TB/HIV co-infected and 465 non-co-infected HIV patients. CMD was assessed through face to face interviews by trained clinical nurses using the Kessler 10 scale. Several risk factors for CMD were assessed using a structured questionnaire.

**Results-:**

TB/HIV co-infected patients had significantly (p = 0.001) greater risk of CMD (63.7%) than the non-co-infected patients (46.7%). When adjusted for the effect of potential confounding variables, the odds of having CMD for TB/HIV co-infected individuals was 1.7 times the odds for non-co-infected patients [OR = 1.7, (95%CI: 1.0, 2.9)]. Individuals who had no source of income [OR = 1.7, (95%CI: 1.1, 2.8)], and day labourers [OR = 2.4, 95%CI: 1.2, 5.1)] were more likely to have CMD as compared to individuals who had a source of income and government employees respectively. Patients who perceived stigma [OR = 2.2, 95%CI: 1.5, 3.2)] and who rate their general health as "poor" [OR = 10.0, 95%CI: 2.8, 35.1)] had significantly greater risk of CMD than individual who did not perceive stigma or who perceived their general health to be "good".

**Conclusion-:**

TB/HIV control programs should develop guidelines to screen and treat CMD among TB/HIV co-infected patients. Screening programs should focus on individuals with no source of income, jobless people and day labourers.

## Background

The global burden of disease report revealed that neuropsychiatric conditions accounted for up to a quarter of all the disability-adjusted life years lost[1]. In low and middle income countries (LAMIC), neuropsychiatric disorders such as depression, anxiety and somatoform disorders account for 9.8% of the global burden of diseases[2].

The risk factors for mental health problems are complex [3]. Poverty, low education, social exclusion, gender disadvantage, conflict and disasters are the major social determinants of mental disorders[2]. Presence of medical illnesses [4] stigma and discrimination [5] also play a role in the development of depression.

The recent Lancet series on global mental health highlighted the lack of research on the interactions between mental disorders and communicable diseases such as tuberculosis (TB) and HIV/AIDS in low income settings [4]. A high rate of depressive disorders has been reported among patients with HIV in high income countries. In the United States, a higher rate of depressive disorders was observed in HIV seropositive women (19%) than in HIV seronegative women (4.8%)[6]. Bing and colleagues [7] also reported that the 12-month prevalence of major depression was 36% in HIV infected adults compared to 7.6% in the general population in the United States. Some studies in LAMIC (Kenya, Democratic Republic of Congo and Thailand) also revealed that the rate of depression was higher in HIV seropositive patients than in HIV negative individuals [8].

The neurotropism of the HIV, opportunistic infections of the central nervous system and side effects of antiretroviral therapy (ART), especially the Efavirenz, may cause mental health problems[4]. There is evidence that individuals with mental health problems are at higher risk of contracting HIV due to their higher risk behaviours[9].

Adherence to ART is adversely affected by depression[10-12]. Moreover, depression may reduce the CD4 lymphocyte count, increase viral load and mortality in patients with AIDS[13-15]. On the other hand, treatment of depression has been found to improve health outcomes in patients with HIV[14,15].

Some studies have investigated the relationship between common mental disorders (CMD) and TB. A study conducted in Turkey showed that the prevalence of depression and anxiety was 19% for recently diagnosed patients with TB, 22% for defaulted TB patients, and 26% for patients with multidrug-resistant TB[16]. The prevalence of CMD in 53 Nigerian TB patients recruited in a chest clinic was 30%, compared to 5% in healthy controls[17]. A study done in Pakistan showed that the prevalence of depression and anxiety among TB patients was 43% and 47% respectively[18]. TB patients have poor mental health and quality of life compared to the general population in United Kingdom[19].

TB/HIV co-infection poses immense diagnostic and economic challenges for developing countries[20]. HIV is the strongest risk factor for the development of active pulmonary and extra pulmonary TB. TB also accelerates the deterioration of the immune status of patients with HIV [21-24] and it is one of the leading causes of mortality in people living with HIV/AIDS (PLHA)[25]. In Ethiopia, the rate of TB/HIV co-infection ranges from 45% in Addis Ababa[26] to 52% in North Ethiopia[27].

Although several studies have been done concerning the interaction between either HIV/AIDS or TB with mental health problems, little is known about the effect of TB/HIV co-infection on common mental disorders. CMD, characterized by a significant level of depressive, anxiety and/or somatic symptoms are common among women in Ethiopia[28,29]. So far the magnitude of CMD among men and the interaction between CMD and communicable diseases has not been investigated in Ethiopia. The objective of this study was to compare the occurrence of CMD in TB/HIV co-infected and non-co-infected HIV patients in Ethiopia.

## Methods

### Study Settings and Population

From February to March, 2009, we conducted a cross sectional study in three hospitals in Oromiya regional state of Ethiopia. Based on the availability of patients, we selected Adama, Nekemet and Jimma specialized hospitals in the east, west and southwest part of Ethiopia respectively. The methodology of this study is described elsewhere[30]. In brief, the study population consisted of 155 TB/HIV co-infected and 465 non-co-infected HIV patients. All TB patients in the first two months of TB treatment were asked to participate in the study. For each TB/HIV co-infected patient, 3 non-co-infected HIV patients were also asked to participate. The latter were selected using a simple random sampling technique using the patients' unique identification number in the HIV clinics. TB was diagnosed using national TB guidelines[31]. The non-co-infected patients were also screened for the presence of any signs and symptoms of TB. Non-co-infected HIV patients with a prior history of TB were excluded from the study. Patients who were less than 15 years old, had TB meningitis or another opportunistic infection, had a chronic illness like diabetes, cardiovascular disease or hypertension were excluded from the study.

### Data collection procedures

CMD was assessed through face to face interviews by trained clinical nurses using the Kessler 10 scale[32] which consisted of 10 five-point Likert scale (0 = never, 1 = rarely, 2 = some of the time, 3 = most of the time, 4 = all of the time) questions. For the diagnosis of CMD, the Kessler 10 scale was validated in Ethiopia against a gold standard of psychiatrists' diagnosis[28]. In a previous study, two psychiatrists used the validated Comprehensive Psychopathology Rating Scale (CPRS) to diagnose CMD[29]. The CPRS has 66 items; 40 symptoms based on the subjective report of the interviewee, 25 signs rated on the basis of observation during the interview and a global rating indicating presence of significant mental disorder. The presence of clearly defined symptoms or signs of mental disorder was rated on a 4-point scale (0-3). The definitions of each scale point were standardized as follows: 0 = not present; 1 = doubtful whether present, and not interfering with life; 2 = definitely present and of moderate severity; 3 = severe or incapacitating. The psychiatrists were asked to conduct a full psychiatric interview and then complete the CPRS ratings using all available information[28,29]. Caseness of CMD was determined on the basis of any of the combination of depressive, anxiety and/or somatic symptoms present at clinically significant level. At the cut-off point of 6/7, the Kessler 10 scale had a sensitivity and specificity of 84.2% and 77.8% respectively to diagnose CMD[28].

Perceived stigma was measured using a questionnaire adopted from Berger et al[33]. The instrument was highly reliable in a pre-test (Cronbach's α = 0.93). The stigma scale consisted of four-point Likert scale (strongly disagree, disagree, agree, strongly agree) questions concerning perceived isolation, shame, guilt and disclosure of the HIV status. Item scores of the stigma questions were summed to construct a single stigma variable. Participants were classified as having or not having perceived stigma using the mean of the stigma variable as cut-off point. Perceived general health of the participants (good, medium and poor) was assessed by asking the question 'How would you rate your health?'

Medical charts were reviewed to collect clinical information and level of adherence to antiretroviral and TB treatment. Individuals who took more than 95% of the prescribed doses were labelled as adherent to antiretroviral therapy.

### Data Analysis

Data were analyzed using SPSS version 16.0 software. Item scores of the Kessler scale were summed and individuals who scored above the cut-off point of 6/7 [28]were labelled as having CMD. The Pearson's chi-square test was used to evaluate the association between exposure variables (TB/HIV co-infection, socio-demographic and clinical characteristics of the patients) and CMD. A stepwise logistic regression model was used to adjust for the effect of confounding variables. Variables significantly associated (P < 0.05) with CMD or TB/HIV co-infection in the Pearson's chi-square test were included in the logistic regression model.

### Ethical consideration

Ethical clearance was obtained from the Jimma University ethical review board. Written informed consent was obtained from the study participants. To ensure confidentiality, we used codes to analyze the data.

## Results

All of the 465 non-co-infected HIV patients and 124 (80%) of the co-infected HIV patients participated in the study; 31 TB/HIV co-infected patients were lost to follow up before interview. Of the co-infected patients, smear negative, smear positive and extra pulmonary TB accounted for 61 (49.2%), 42 (33.9%), 21 (16.9%) respectively. From the total TB/HIV co-infected patients, 4 (3.2%) interrupted their TB treatment once.

The age distribution of TB/HIV co-infected and non-co-infected patients was similar. Fifty percent of the co-infected and 60.2% of the non-co-infected HIV patients were females (P < 0.05). Higher proportion of co-infected patients (43.5%) had no source of income compared to the non-co-infected patients (26.7%) [OR = 1.9, (95%CI: 1.0, 3.8)]. After adjusting for confounding variables, co-infected patients (57.5%) were more likely to have a lower CD4 lymphocyte count than non-co-infected patients (27.4%) [OR = 3.6; (95%CI: 2.1, 6.1)]. Before the diagnosis of TB, significantly larger proportions (78.0%) of the co-infected patients were in stage III of the WHO classification compared to the non-co-infected HIV patients (56.0%). All of the non-co-infected and 75.6% of the co-infected patients were taking antiretroviral treatment during the survey (Table-1).

**Table 1:** Socio-demographic and clinical characteristics of the study population in three hospitals of, Ethiopia, April, 2009

Variables	TB/HIV coinfected patients	HIV patients	Crude OR	Adjusted OR
	Number (%)	Number (%)	(95%CI)	(95%CI)
**Age in Years**				
15-24	12(9.7)	42(9.1)	1	-
25-34	53(42.7)	221(47.5)	0.8(0.4,1.7)	
> = 35	59(47.6)	202(43.4)	1.0(0.5,2.1)	
**Sex**				
Male	62(50)	185(39.8)	1.5(1.0, 2.2)*	1.9(1.0,3.7)*
Female	62(50)	280(60.2)	1	1
**Educational status**				
Illiterate	30(24.2)	74(15.9)	1	1
literate	94(75.8)	391(84.1)	0.6(0.4, 0.9)*	0.8(0.4,1.6)
**Marital status**				
Single	24(19.3)	75(16.1)	1.3(0.7,2.5)	-
Married	42(33.9)	191(41.1)	0.9(0.5,1.6)	
Divorced	33(26.6)	93(20.0)	1.5(0.8,2.7)	
Widowed	25(20.2)	106(22.8)	1	
**Occupation (n = 580)**				
Government worker	17(14.8)	51(11.0)	1	1
Private employee	15(13.1)	83(17.8)	0.5(0.2,1.1)	0.9(0.3,2.7)
Merchant	1(0.9)	69(14.8)	0.4(0.2,1.0)	0.6(0.2,2.3)
Farmer	13(11.3)	32(6.9)	1.2(0.5,2.8)	2.0(0.6,6.5)
Housewives	15(13.0)	79(17.0)	0.6(0.3,1.3)	1.4(0.4,4.7)
Day laborers	22(19.1)	93(20.0)	0.7(0.4,1.5)	1.6(0.6,4.8)
No Job	32(27.8)	58(12.5)	1.6(1.1, 3.4)*	1.7(0.5,5.1)
**Have source of income**				
Yes	70(56.5)	341(73.3)	1	1
No	54(43.5)	124(26.7)	2.2(1.4,3.2)*	1.9(1.0,3.8)*
**WHO staging(n = 582)**				
Stage II	13(10.6)	136(29.6)	1	1
Stage III	96(78.0)	257(56.0)	1.6(1.3,1.9)*	2.3(1.1,4.6)*
Stage IV	14(11.4)	66(14.4)	1.2(0.9,1.5)	1.3(0.5,3.5)
**CD4 lymphocyte count(n = 489)**				
< 200	46(57.5)	112(27.4)	1.9(1.2,2.8)*	3.6(2.1,6.1)*
> = 200	34(42.5)	297(72.6)	1	1
**Antiretroviral therapy**				
Yes	93(75.6)	462(100)	0.02(0.001, 0.04)	0.01(0.001, 0.003)*
No	31(24.4)	3	1	

*statistically significant (P < 0.05)

The internal consistency of the Kessler 10 scale was high (Cronbach's α = 0.93) and correlation between items in the Kessler scale ranged from 0.50 to 0.79.

A lower CD4 lymphocyte count, a WHO stage of III and IV, taking antiretroviral treatment, types of antiretroviral therapy, adherence to antiretroviral therapy, age, sex, literacy and marital status were not significantly associated with CMD in the bivariate analysis. On the other hand, occupation, source of income, TB/HIV co-infection, perceived stigma and perceived general health were significantly associated with CMD in the bivariate analysis and were therefore further evaluated in the multivariable model. Individuals who had no source of income [OR = 1.7, (95%CI: 1.1, 2.8)], and day laborers [OR = 2.4, 95%CI: 1.2, 5.1)] were more likely to have CMD as compared to individuals who had a source of income and government employees respectively. CMD was present in 63.7% of the TB/HIV co-infected patients and in 46.7% of the non-co-infected patients. TB/HIV co-infected patients were 1.7 times more likely to experience CMD than the non-co-infected patients [OR = 1.7, (95%CI: 1.1, 2.9)]. Patients who perceived stigma [OR = 2.2, 95%CI: 1.5, 3.2)] and who rate their general health as "poor" [OR = 10.0, 95%CI: 2.8, 35.1)] had significantly greater risk of CMD than individual who did not perceive stigma or who perceived their general health to be "good" (Table-2).

**Table 2:** Association of socio-demographic and clinical characteristics and CMD in three hospitals of Ethiopia

Variables	Common Mental Disorders		Crude OR	Adjusted OR
			
	Yes (%)	No (%)	(95%CI)	(95%CI)
**Sex**				
Male	132(53.4)	115(46.6)	1	
Female	164(48.0)	178(52.0)	0.8(0.5,1.1)	-
**Literacy status**				
Literate	236(48.7)	249(51.3)	1	-
Non-literate	60(57.7)	44(42.3)	1.4(0.9,2.2)	
**Age**				
15-24	21(38.9)	33(61.1)	1	-
25-35	145(52.9)	129(47.1)	1.7(0.9,3.2)	
> = 35	130(49.8)	131(50.2)	1.5(0.8,2.8)	
**Marital status**				
Single	48(48.5)	51(51.5)	0.6(0.4,1.0)	-
Married	106(45.5)	127(54.5)	0.5(0.4,0.9)	
Divorced	65(51.6)	61(48.4)	0.8(0.5,1.2)	
Widowed	77(58.8)	54(41.2)	1	
**Occupation**				
Government employee	23(33.8)	45(66.2)	1	1
Private employee	49(50.0)	49(50.0)	1.9(1.0, 3.7) *	2.2 (1.1,4.6)*
Merchant	35(44.3)	44(55.7)	1.5(0.8,3.0)	2.1 (1.0,4.4)*
Farmers	24(53.3)	21(46.7)	2.2(1.0, 4.8) *	1.8 (0.8,4.2)
Housewives	40(42.6)	54(57.4)	1.4(0.7,2.7)	1.2 (0.5,2.8)
Day Laborers	66(57.9)	48(42.1)	2.7(1.4, 5.0) *	2.4 (1.2, 5.1) *
Jobless	59(64.8)	32(35.2)	3.6(1.8, 6.9) *	2.5 (1.1,5.6) *
**Have source of income**				
Yes	190(46.2)	221(53.8)	1	1
No	106(59.3)	72(40.7)	1.6(1.2,2.4) *	1.7(1.1,2.8) *
**HIV/TB co-infection**				
No	217(46.7)	248(53.3)	1	1
Yes	79(63.7)	45(36.3)	2.0(1.3,3.0) *	1.7 (1.1,2.9) *
**WHO staging(n = 582)**				
Stage II	73(49)	76(51)	1	
Stage III	194(55.0)	159(45.0)	1.1(0.5, 1.2)	-
Stage IV	24(30)	56(70)	0.8(0.7, 1.5)	
**CD4 lymphocyte count(n = 489)**				
< 200	74(46.8)	84(53.2)	1.2(0.8.1.7)	-
> = 200	171(51.7)	160(48.3)	1	
**Taking antiretroviral therapy**				
Yes	274(49.4)	281(50.6)	1	-
No	22(64.7)	12(35.3)	1.8(0.9,3.8)	
**Adherence to antiretroviral therapy(ART)**				
Yes	285(49.7)	288(50.3)	1	-
No	8(50)	8(50)	1.1(0.3,2.7)	
**ART regimen(n = 555)**				
Nevirapine based	179(48.5)	190(51.5)	1	
Efavirenz based	95(51.1)	91(48.9)	1.1(0.7,1.5)	-
**Perceived Stigma**				
No	132(41.9)	183(58.1)	1	1
Yes	164(59.9)	110(40.1)	2.0(1.4,2.8)*	2.2(1.5,3.2)*
**Perceived General Health**				
Good	168(39.4)	258(60.6)	1	1
Medium	102(76.1)	32(23.9)	4.8(3.1.7.6)*	4.7 (2.9,7.6) *
Poor	26(89.7)	3(10.3)	13.3(3.9,44.5)*	10.0 (2.8,35.1)*

*P-Value < 0.05

## Discussion

We assessed the occurrence of CMD in TB/HIV co-infected and non-co-infected patients using the Kessler scale (K10)[28]. Caseness of CMD was assessed using a cut-off point of 6/7 of the K10 which had a sensitivity and specificity of specificity of 84.2% and 77.8% against the psychiatrists' assessment using CPRS. Several studies showed that the K10 had strong psychometric properties[32,34-36] and can discriminate between cases and non-cases reported in the Diagnostic and Statistical Manual of Mental Disorders (DSM-IV)[32,36]. In this study, 79 (64%) of the TB/HIV co-infected patients had CMD which is higher than the findings of Hanlon et al (33%) [28] and Tesfaye et al (40%)[28] among women in Ethiopia. The prevalence of depression among HIV seropositive women (19%) in the United States was also lower as compared to our study[6]. The differences in prevalence of mental disorders could be attributable to several factors including the population being studied, the study periods and the diagnostic tools[29]. TB/HIV co-infected patients were 1.7 times more likely to have CMD than non-co-infected HIV patients. However, we did not find a significant association between CD4 lymphocyte count and CMD, which is consistent with several previous studies[37-39]. TB/HIV co-infected patients can be at higher risk of CMD as a result of stigma and discrimination by the society[5]. We also found out that perceived stigma was one of the independent predictors of CMD. People with perceived stigma may have a low self image and be socially isolated which may predispose them to CMD[40].

Poverty is known to be a major risk factor for mental health problems [3]. Many individuals in developing countries suffer from CMD as a result of stress caused by poverty[3,41]. In this study, daily labourers and jobless individuals, both economically disadvantaged groups, were at a higher risk of CMD. Use of the locally made alcohol called "Katikala", which is common among daily labourers in Ethiopia [42], can also predispose to CMD. In contrast with other studies[3,40,43], we did not observe a significant association between marital status, gender and CMD.

In our study, the perception of poor general health was strongly associated with CMD. However, there could be a bi-directional relationship between perceived general health and depression. Poor perceived general health could have resulted from the presence of CMD.

Although antiretroviral drugs, particularly Efavirenz, can cause CMD[4], our result did not show an association between this drug and CMD. Adherence to antiretroviral therapy has been reported to be affected by depression[4]. However, in our study we did not observe an association between adherence and CMD.

Some methodological limitations need to be noted in interpreting the findings of this study. First, the Kessler 10 scale is not 100% sensitive and specific and we might have misdiagnosed or missed some cases of CMD. Second, no detailed validation study was done for the stigma scale. Third, 20% of the TB/HIV co-infected patients were lost to follow-up which might introduce information bias. Important risk factors of CMD such as substance and alcohol use were not assessed.

## Conclusions

TB/HIV co-infected patients were at higher risk of developing CMD than non-co-infected patients. Occupation, perceived stigma and perceived general health were other risk factors for CMD. TB/HIV control programs should develop guidelines to screen and treat CMD among TB/HIV co-infected patients. Screening programs should focus on individuals with no source of income, jobless people and day labourers. We recommend prospective cohort study to investigate the cause effect relationship of risk factors and CMD.

## Competing interests

The authors declare that they have no competing interests.

## Authors' contributions

**AD **conceived and designed the study, analyzed the data and drafted the manuscript. **MT **participated in the design, conception and reviewed the article. **YH, GA **and **LA **were involved in report writing and reviewing. **LD **and **RC **were involved in analysis and critically reviewed the article. All authors read and approved the final manuscript.

## Pre-publication history

The pre-publication history for this paper can be accessed here:

http://www.biomedcentral.com/1471-2334/10/201/prepub

## References

[B1] Lopez AD Mathers CD Ezatti M Jamison D Murray C Global burden of diseases and risk factors 2006 New York, Oxford University Press

[B2] Patel V Mental health in low- and middle-income countries Br Med Bull 2007 81-82 81 96 10.1093/bmb/ldm010 17470476

[B3] WHO Mental Health, New understanding, new hope: The world health report, WHO, 2001 http://www.who.int/whr/2001/en/

[B4] Prince M Patel V Saxena S Maj M Maselko J Phillips MR Rahman A No health without mental health Lancet 2007 370 9590 859 877 10.1016/S0140-6736(07)61238-0 17804063

[B5] Li L Lee SJ Thammawijaya P Jiraphongsa C Rotheram-Borus MJ Stigma, social support, and depression among people living with HIV in Thailand AIDS Care 2009 21 8 1007 1013 10.1080/09540120802614358 20024757 PMC2803757

[B6] Morrison MF Petitto JM Ten Have T Gettes DR Chiappini MS Weber AL Brinker-Spence P Bauer RM Douglas SD Evans DL Depressive and anxiety disorders in women with HIV infection Am J Psychiatry 2002 159 5 789 796 10.1176/appi.ajp.159.5.789 11986133

[B7] Bing EG Burnam MA Longshore D Fleishman JA Sherbourne CD London AS Turner BJ Eggan F Beckman R Vitiello B Psychiatric disorders and drug use among human immunodeficiency virus-infected adults in the United States Arch Gen Psychiatry 2001 58 8 721 728 10.1001/archpsyc.58.8.721 11483137

[B8] Maj M Janssen R Starace F Zaudig M Satz P Sughondhabirom B Luabeya MA Riedel R Ndetei D Calil HM WHO Neuropsychiatric AIDS study, cross-sectional phase I. Study design and psychiatric findings Arch Gen Psychiatry 1994 51 1 39 49 8279928 10.1001/archpsyc.1994.03950010039006

[B9] Koblin BA Husnik MJ Colfax G Huang Y Madison M Mayer K Barresi PJ Coates TJ Chesney MA Buchbinder S Risk factors for HIV infection among men who have sex with men Aids 2006 20 5 731 739 10.1097/01.aids.0000216374.61442.55 16514304

[B10] Paterson DL Swindells S Mohr J Brester M Vergis EN Squier C Wagener MM Singh N Adherence to protease inhibitor therapy and outcomes in patients with HIV infection Ann Intern Med 2000 133 1 21 30 10877736 10.7326/0003-4819-133-1-200007040-00004

[B11] Ammassari A Antinori A Aloisi MS Trotta MP Murri R Bartoli L Monforte AD Wu AW Starace F Depressive symptoms, neurocognitive impairment, and adherence to highly active antiretroviral therapy among HIV-infected persons Psychosomatics 2004 45 5 394 402 10.1176/appi.psy.45.5.394 15345784

[B12] Gordillo V del Amo J Soriano V Gonzalez-Lahoz J Sociodemographic and psychological variables influencing adherence to antiretroviral therapy Aids 1999 13 13 1763 1769 10.1097/00002030-199909100-00021 10509579

[B13] Leserman J Role of depression, stress, and trauma in HIV disease progression Psychosom Med 2008 70 5 539 545 10.1097/PSY.0b013e3181777a5f 18519880

[B14] Ironson G O'Cleirigh C Fletcher MA Laurenceau JP Balbin E Klimas N Schneiderman N Solomon G Psychosocial factors predict CD4 and viral load change in men and women with human immunodeficiency virus in the era of highly active antiretroviral treatment Psychosom Med 2005 67 6 1013 1021 10.1097/01.psy.0000188569.58998.c8 16314608 PMC2614887

[B15] Ickovics JR Hamburger ME Vlahov D Schoenbaum EE Schuman P Boland RJ Moore J Mortality, CD4 cell count decline, and depressive symptoms among HIV-seropositive women: longitudinal analysis from the HIV Epidemiology Research Study Jama 2001 285 11 1466 1474 10.1001/jama.285.11.1466 11255423

[B16] Aydin IO Ulusahin A Depression, anxiety comorbidity, and disability in tuberculosis and chronic obstructive pulmonary disease patients: applicability of GHQ-12 Gen Hosp Psychiatry 2001 23 2 77 83 10.1016/S0163-8343(01)00116-5 11313075

[B17] Aghanwa HS Erhabor GE Demographic/socioeconomic factors in mental disorders associated with tuberculosis in southwest Nigeria J Psychosom Res 1998 45 4 353 360 10.1016/S0022-3999(98)00006-3 9794281

[B18] Husain MO Dearman SP Chaudhry IB Rizvi N Waheed W The relationship between anxiety, depression and illness perception in tberculosis patients in Pakistan Clin Pract Epidemiol Ment Health 2008 4 4 10.1186/1745-0179-4-4 18302758 PMC2288599

[B19] Kruijshaar ME Lipman M Essink-Bot ML Lozewicz S Creer D Dart S Maguire H Abubakar I Health status of UK patients with active tuberculosis Int J Tuberc Lung Dis 14 3 296 302 20132620

[B20] WHO Global Tuberculosis control: Surveillance, planning and Financing. World Report 2008 http://www.who.int/tb/publications/global_report/en/

[B21] Aliyu MH Salihu HM Tuberculosis and HIV disease: two decades of a dual epidemic Wien Klin Wochenschr 2003 115 19-20 685 697 10.1007/BF03040884 14650943

[B22] Maher D Watt CJ Williams BG Raviglione M Dye C Tuberculosis deaths in countries with high HIV prevalence: what is their use as an indicator in tuberculosis programme monitoring and epidemiological surveillance? Int J Tuberc Lung Dis 2005 9 2 123 127 15732729

[B23] Harries AD Hargreaves NJ Kemp J Jindani A Enarson DA Maher D Salaniponi FM Deaths from tuberculosis in sub-Saharan African countries with a high prevalence of HIV-1 Lancet 2001 357 9267 1519 1523 10.1016/S0140-6736(00)04639-0 11377627

[B24] Mukadi YD Maher D Harries A Tuberculosis case fatality rates in high HIV prevalence populations in sub-Saharan Africa Aids 2001 15 2 143 152 10.1097/00002030-200101260-00002 11216921

[B25] WHO Stop TB Department and Department of HIV/AIDS. Interim policy on collaborative TB/HIV activities. WHO/HTM/TB/2004.330 2004

[B26] Demissie MLB Tegbaru B Human Immunodeficiency virus (HIV) infection in tuberculosis patients in Addis Ababa. Ethiop 2000 14 277 282

[B27] Kassu A Mengistu G Ayele B Diro E Mekonnen F Ketema D Moges F Mesfin T Getachew A Ergicho B Coinfection and clinical manifestations of tuberculosis in human immunodeficiency virus-infected and -uninfected adults at a teaching hospital, northwest Ethiopia J Microbiol Immunol Infect 2007 40 2 116 122 17446959

[B28] Tesfaye M Hanlon C Wondimagegn D Alem A Detecting postnatal common mental disorders in Addis Ababa, Ethiopia: validation of the Edinburgh Postnatal Depression Scale and Kessler Scales J Affect Disord 122 1-2 102 108 10.1016/j.jad.2009.06.020 19615753

[B29] Hanlon C Medhin G Alem A Araya M Abdulahi A Hughes M Tesfaye M Wondimagegn D Patel V Prince M Detecting perinatal common mental disorders in Ethiopia: validation of the self-reporting questionnaire and Edinburgh Postnatal Depression Scale J Affect Disord 2008 108 3 251 262 10.1016/j.jad.2007.10.023 18055019

[B30] Deribew A Tesfaye M Hailmichael Y Negussu N Daba S Wogi A Belachew T Apers L Colebunders R Tuberculosis and HIV co-infection: its impact on quality of life Health Qual Life Outcomes 2009 7 105 10.1186/1477-7525-7-105 20040090 PMC2809048

[B31] Ministry of Health, Ethiopia TB, leprosy and TB/HIV prevention and control program, Manual. Addis Ababa, Ethiopia 2008 http://www.moh.gov.et/

[B32] Kessler RC Andrews G Colpe LJ Hiripi E Mroczek DK Normand SL Walters EE Zaslavsky AM Short screening scales to monitor population prevalences and trends in non-specific psychological distress Psychol Med 2002 32 6 959 976 10.1017/S0033291702006074 12214795

[B33] Berger BE Ferrans CE Lashley FR Measuring stigma in people with HIV: psychometric assessment of the HIV stigma scale Res Nurs Health 2001 24 6 518 529 10.1002/nur.10011 11746080

[B34] Donker T Comijs H Cuijpers P Terluin B Nolen W Zitman F Penninx B The validity of the Dutch K10 and extended K10 screening scales for depressive and anxiety disorders Psychiatry Res 176 1 45 50 10.1016/j.psychres.2009.01.012 20071036

[B35] Fassaert T De Wit MA Tuinebreijer WC Wouters H Verhoeff AP Beekman AT Dekker J Psychometric properties of an interviewer-administered version of the Kessler Psychological Distress scale (K10) among Dutch, Moroccan and Turkish respondents Int J Methods Psychiatr Res 2009 18 3 159 168 10.1002/mpr.288 19701920 PMC6878421

[B36] Furukawa TA Kessler RC Slade T Andrews G The performance of the K6 and K10 screening scales for psychological distress in the Australian National Survey of Mental Health and Well-Being Psychol Med 2003 33 2 357 362 10.1017/S0033291702006700 12622315

[B37] Evans DL Ten Have TR Douglas SD Gettes DR Morrison M Chiappini MS Brinker-Spence P Job C Mercer DE Wang YL Association of depression with viral load, CD8 T lymphocytes, and natural killer cells in women with HIV infection Am J Psychiatry 2002 159 10 1752 1759 10.1176/appi.ajp.159.10.1752 12359683

[B38] Perry S Fishman B Jacobsberg L Frances A Relationships over 1 year between lymphocyte subsets and psychosocial variables among adults with infection by human immunodeficiency virus Arch Gen Psychiatry 1992 49 5 396 401 1586275 10.1001/archpsyc.1992.01820050060010

[B39] Rabkin JG Williams JB Remien RH Goetz R Kertzner R Gorman JM Depression, distress, lymphocyte subsets, and human immunodeficiency virus symptoms on two occasions in HIV-positive homosexual men Arch Gen Psychiatry 1991 48 2 111 119 1671196 10.1001/archpsyc.1991.01810260019002

[B40] Perlick DA Rosenheck RA Clarkin JF Sirey JA Salahi J Struening EL Link BG Stigma as a barrier to recovery: Adverse effects of perceived stigma on social adaptation of persons diagnosed with bipolar affective disorder Psychiatr Serv 2001 52 12 1627 1632 10.1176/appi.ps.52.12.1627 11726754

[B41] Araya R Rojas G Fritsch R Acuna J Lewis G Common mental disorders in Santiago, Chile: prevalence and socio-demographic correlates Br J Psychiatry 2001 178 228 233 10.1192/bjp.178.3.228 11230033

[B42] Deribew A Distribution of Most-at-risk Population Groups and their perceptions towards HIV/AIDS: A Baseline Survey in Amhara Region for the Implementation of Mobile HIV Counselling and Testing 2009 Bethesda, MD: Private Sector Program-Ethiopia, Abt Associates Inc

[B43] Seedat S Scott KM Angermeyer MC Berglund P Bromet EJ Brugha TS Demyttenaere K de Girolamo G Haro JM Jin R Cross-national associations between gender and mental disorders in the World Health Organization World Mental Health Surveys Arch Gen Psychiatry 2009 66 7 785 795 10.1001/archgenpsychiatry.2009.36 19581570 PMC2810067

